# Cytomegalovirus-Induced Hemophagocytic Lymphohistiocytosis in an Immunocompromised Patient with Inflammatory Bowel Disease

**DOI:** 10.1155/2024/6964818

**Published:** 2024-04-02

**Authors:** Alessandro Pedicelli, René P. Michel, Nick Krassakopoulos

**Affiliations:** ^1^Division of Internal Medicine, Department of Medicine, McGill University, Montreal, QC, Canada; ^2^Department of Pathology, McGill University, Montreal, QC, Canada

## Abstract

Hemophagocytic lymphohistiocytosis (HLH) is a rare and often fatal syndrome of immune hyperactivation, cytokine dysregulation, and severe inflammation. This severe syndrome is commonly triggered by infection, malignancy, autoimmunity, or immunosuppression. We present herein the case of a 56-year-old-female diagnosed with HLH triggered by an acute cytomegalovirus (CMV) infection with viremia in the context of immunosuppression for inflammatory bowel disease. This case highlights the importance of utilizing multiple diagnostic tools, prompt initiation of anti-hemophagocytic treatment, and management of the underlying etiology, to prevent significant morbidity and mortality.

## 1. Introduction

Hemophagocytic lymphohistiocytosis (HLH) is a rare and often fatal diagnosis characterized by immune hyperactivation, cytokine dysregulation, and severe inflammation. With an estimated incidence of 1.2 cases per million per year and a mortality rate of 47%, the early recognition and diagnosis of this syndrome is crucial in preventing significant morbidity and mortality [1]. HLH is traditionally classified as either primary or secondary. Primary, or familial HLH, is caused by inherited genetic mutations that disrupt the function of cytotoxic T cells and natural killer (NK) cells [2]. Symptoms are often severe and present early in life [3]. Secondary or acquired HLH is induced by a variety of triggers including infection, malignancy, autoimmunity, and immunosuppression [2, 3]. The diagnosis of HLH is established by the presence of five or more of eight diagnostic criteria defined by the HLH-2004 consensus [3, 4]. These include (1) fever, (2) splenomegaly, (3) cytopenias affecting at least two lineages in the peripheral blood, (4) hypertriglyceridemia and/or hypofibrinogenemia, (5) hemophagocytosis seen in bone marrow, spleen, or lymph nodes, (6) low or absent NK-cell activity, (7) ferritin ≥500 *μ*g/L, and (8) soluble IL-2 receptor/CD25 ≥ 2400 U/mL [4]. Another useful diagnostic tool is the HScore, which estimates the probability of a reactive hemophagocytic syndrome based on a variety of clinical and biochemical factors [5]. Although Epstein–Barr virus (EBV) remains the most common infectious etiology associated with secondary HLH, cytomegalovirus (CMV) is an important trigger for the syndrome, especially in patients with autoimmune disease treated with immunosuppressive medications [2]. A recent systematic review by Rolsdorph et al. [3] identified 74 published cases of CMV-related HLH in the literature. Interestingly, the most common comorbidity identified in these patients was inflammatory bowel disease, which was reported in 30% of cases identified.

## 2. Case Presentation

Our patient is a 56-year-old female who presented to a community hospital with a three-week history of vague generalized weakness, fatigue, subjective fevers and chills, mild dyspnea on exertion, and night sweats. She also described anorexia and a 25 lb weight loss over the last three weeks. Her past medical and surgical history revealed Crohn disease treated with azathioprine and infliximab, hypertension, and a remote nasal septoplasty. She had no allergies, did not drink alcohol, or smoke or use drugs.

**Figure 1:**
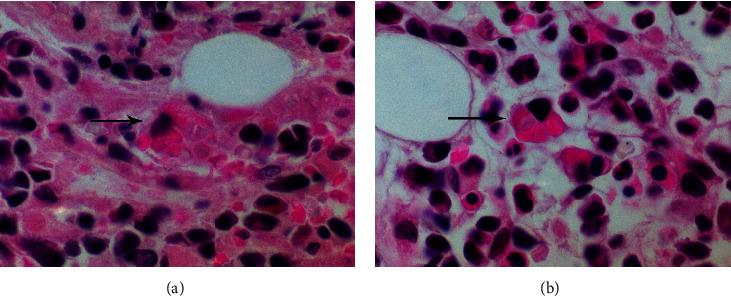
(a, b) High-power micrographs taken of the first bone marrow core biopsy done at the community hospital. Note: macrophages with ingested erythrocytes (arrows).

Upon presentation to the emergency room, she was found to be intermittently febrile at 39-40°C with all other vital signs within normal limits. The physical exam revealed mildly decreased air entry at the bases of both lungs but otherwise noncontributory. Initial laboratory results revealed pancytopenia, hyperferritinemia, hypertriglyceridemia, and hypofibrinogenemia (see Table 1 for detailed results). Computed tomography imaging of the chest, abdomen, and pelvis showed bilateral pulmonary ground glass opacities (GGOs) suspicious for infection, without splenomegaly or any evidence of malignancy. Initial infectious workup was negative for a multiplex respiratory virus PCR panel, *Legionella*, *C. difficile*, hepatitis B and C, and malaria. Her preliminary blood and urine cultures were all negative. CMV, EBV, HIV, and fungal infection markers were also sent but had not yet resulted. Given the high suspicion for HLH (at the time, the patient met four of eight of diagnostic criteria and an HScore of 244 (99% probability of hemophagocytic syndrome), see Table 1 for details), the patient underwent a bone marrow aspirate and biopsy, which revealed a normocellular marrow with rare cells exhibiting hemophagocytosis (see Figure 1). However, it was the opinion of the treating hematologist that the bone marrow aspirate evidence was not fully convincing of a reactive hemophagocytic syndrome.

Despite treatment with broad-spectrum antibiotics (piperacillin-tazobactam 4.5 g IV Q6H plus vancomycin 1.5 g IV Q12H) and dexamethasone 15 mg IV daily, the patient remained febrile with an increasing serum ferritin level, and she was transferred to our care centre for further workup and management.

Upon arrival at our centre, the patient remained intermittently febrile and underwent another bone marrow biopsy; a broadened infectious workup was sent. The bone marrow biopsy results and analysis showed a few cells with hemophagocytosis, confirming the diagnosis of HLH. The patient was then found to have CMV viremia, with a quantitative serum CMV PCR showing over 2 million copies/mL. Treatment with ganciclovir 275 mg IV Q12H was promptly initiated. Subsequent ophthalmologic evaluation revealed CMV retinitis. Further workup of the bilateral lung opacities with bronchoalveolar lavage revealed an acute aspergillus infection, treated with voriconazole 200 mg PO daily and later isavuconazole 200 mg PO daily.

**Figure 2:**
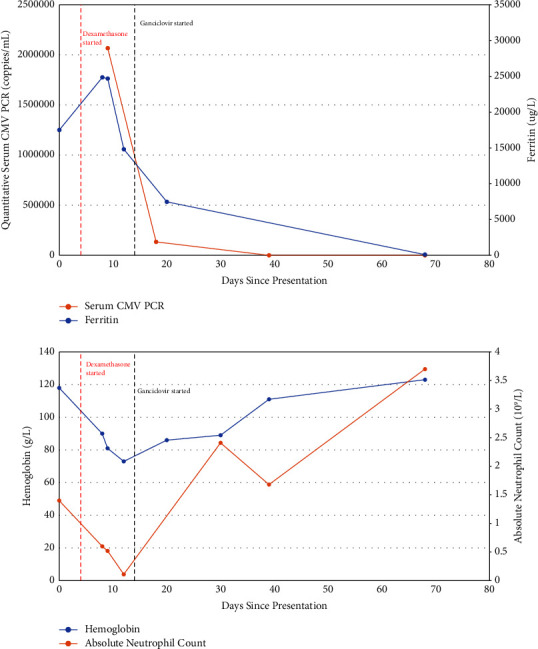
Pertinent biochemical results over the course of hospitalization.

Over the course of the next few weeks, with continued treatment with dexamethasone and antiviral medications, the patient's clinical status improved significantly. She did not require escalation of HLH therapy with antineoplastic or immunosuppressive agents (e.g., etoposide and cyclosporine). She remained afebrile with improving pancytopenia, normalizing ferritin, and decreasing CMV serum viral load (see Figure 2). She was eventually transferred back to her community hospital in stable condition.

Three months after discharge, the patient was seen in follow-up and was clinically well. She remained on valganciclovir and isavuconazole therapy but was able to taper off the dexamethasone. Her symptoms had completely resolved, her CMV viral load was undetectable, and her blood cell counts had normalized.

## 3. Discussion

HLH remains a rare diagnosis with CMV-induced HLH even less common [3]. A 2014 review by Ramos-Casals et al. [1] identified 2197 cases of adult HLH reported in the literature between 1974 and 2011. Among those, viral infections were the most common trigger, although CMV-induced HLH accounted for only 9% of these, which speaks to the distinctiveness of the case presented here.

As indicated above, the prompt recognition, diagnosis, and management of HLH is critical in preventing significant morbidity and possible mortality secondary to widespread and unchecked hyperimmunity. Our case illustrates the utility of multiple complimentary diagnostic tools. The concomitant use of the HLH-2004 consensus guidelines in addition to the validated HScore allowed the treating team to quickly and confidently make the diagnosis of HLH and initiate lifesaving anti-hemophagocytic and antiviral therapies [4, 5].

An interesting aspect of this case is the overlap between CMV-induced HLH and inflammatory bowel disease (IBD). In their systematic review of CMV-associated HLH, Chevalier and colleagues [6] found an overrepresentation of patients with inflammatory bowel disease with the incidence of a hemophagocytic syndrome being highest in patients treated with thiopurine medications, which was the case for our patient. The incidence and prevalence of inflammatory bowel disease in Canada are predicted to continue to rise over the coming years, with an estimated 388 000 Canadians—representing close to 1% of the general population—expected to be suffering from the disease by the year 2030 [7]. Given a prevalence of 70–90% of CMV infections in the general adult population [8], questions that arise are whether we should be paying closer attention to the CMV status of IBD patients, especially those treated with thiopurine medications, and whether we should be screening regularly for reactivation of this virus. Current Canadian guidelines for the management of luminal Crohn's disease and ulcerative colitis do not comment on the role of regular CMV screening in IBD patients [9, 10]. More clinical data pertaining to morbidity and mortality prevention, cost-benefit analysis, and feasibility of CMV screening in IBD are needed before a robust recommendation can be made.

## 4. Conclusion

In summary, hemophagocytic lymphohistiocytosis remains a rare condition associated with malignancy, autoimmunity, immunosuppression, and infection. Cytomegalovirus infection is a particularly rare etiology of HLH, presenting most frequently in immunosuppressed patients, of which patients with inflammatory bowel disease account for a large proportion. Prompt diagnosis of HLH is achieved through use of the HLH-2004 consensus guidelines and the validated HScore. Prompt initiation of anti-hemophagocytic treatment, as well as diagnosis and treatment of the triggering etiology, is crucial in preventing substantial morbidity and mortality. As the prevalence of IBD in Canada continues to increase over the next decade, future research will be needed to assess the role of CMV infection screening in these at-risk patient populations.

## Figures and Tables

**Table 1:** The HLH-2004 consensus diagnostic criteria and the HScore are validated for the screening and diagnosis of HLH.

HLH-2004 diagnostic criteria^4^ (five of eight needed for diagnosis)	Presence in study patient
Fever	Yes, 40°C
Splenomegaly	No
Cytopenia (affecting 2 or more lineages in peripheral blood):	Yes Hemoglobin 83 g/L Neutrophils 0.56 × 10^9^/L
(i) Hemoglobin <90 g/L	
(ii) Platelets <100 × 10^9^/L	
(iii) Neutrophils <1.0 × 10^9^/L	
Hypertriglyceridemia and/or hypofibrinogenemia:	Yes Fibrinogen 0.67 g/L
(i) Fasting triglycerides ≥3.0 mmol/L	
(ii) Fibrinogen ≤1.5 g/L	
Hemophagocytosis in bone marrow, spleen, or lymph nodes	Yes
Low or absent NK-cell activity	Not assessed
Ferritin ≥500 *μ*g/L	Yes
	Ferritin 13106 *μ*g/L
Soluble CD25 ≥ 2400 U/mL	Result pending

Variables assessed in HScore^5^	Presence in study patient

Underlying immunosuppression	Yes
Fever	Yes
Hepatosplenomegaly	No
Cytopenia	Yes
Ferritin	Yes
Hypertriglyceridemia	No
Hypofibrinogenemia	Yes
Elevated aspartate aminotransferase	Yes
Hemophagocytosis features on bone marrow aspirate	Yes

The superscripts here refer to the number 4 and 5 papers cited in my bibliography. The normal values for these two validated scores were adapted from those works and reproduced in this table.

## Data Availability

The biochemical and laboratory data used to support the findings of this study are available from the corresponding author upon request.
